# Variational free energy as a conceptual framework for understanding habituation in spinal cord stimulation

**DOI:** 10.3389/fpain.2026.1728339

**Published:** 2026-02-18

**Authors:** Alexander Taghva

**Affiliations:** 1 Department of Neurosurgery, University of California, San Diego, CA, United States; 2 Orange County Neurosurgical Associates, Mission Viejo, CA, United States

**Keywords:** habituation, KL divergence, multimodal therapy, prediction error, spinal cord stimulation, variational free energy

## Abstract

**Introduction:**

Habituation, or loss of clinical benefit over time, is a frequent problem in spinal cord stimulation (SCS). Existing programming strategies such as waveform cycling or closed-loop stimulation try to address it, but there is no common conceptual framework. This paper introduces the variational free energy (VFE) principle as a way to think about habituation in terms that connect clinical observation with computational models of the brain.

**Methods:**

We reviewed the core ideas of VFE, as formulated by Friston and colleagues—recognition density, prior, posterior, accuracy, complexity, and Kullback–Leibler divergence—and explained them with simple examples and equations. We then applied these concepts to clinical scenarios in SCS, with reference to existing literature on habituation, predictors of response, and multimodal outcomes.

**Results:**

The VFE framework treats the brain as an inference engine that balances accuracy (explaining sensory data) against complexity (the cost of changing beliefs). In this lens, habituation can occur when stimulation becomes predictable and weakly informative about the hidden state the patient cares about (e.g., bodily safety), so its precision is down-weighted and the system reverts toward the pre-existing pain model. Conversely, “rescue” and closed-loop strategies may work by restoring informative prediction errors and coupling stimulation to meaningful state changes. The asymmetry of KL divergence formalizes why relapse into pain can be easier than sustained relief.

**Discussion:**

VFE does not replace mechanistic models of SCS but offers a simple way to frame habituation that is mathematically grounded yet approachable. It may help clinicians and programmers understand why therapy sometimes fails, and why broader outcome targets and flexible programming approaches may lead to more durable benefit.

## Introduction

Habituation remains a significant challenge in spinal cord stimulation (SCS). It can be defined as a progressive decline in analgesic efficacy despite ongoing delivery of electrical stimulation that initially provided benefit (1). In an analysis by Al-Kaisy et al, of 1,177 SCS patients, 25.2% had explant of their device at 10 years, with loss of efficacy being the reason for explant in 65% of those patients (2). Loss of effect over time may be attributed to several factors, including trial bias or placebo response, suboptimal programming that magnifies short-term response but is unsustainable, or underlying disease progression. However, clinical observations—most notably the recovery of benefit with “rescue” waveforms in chronically implanted patients—suggest that habituation represents a distinct physiological process (3). Several programming strategies have been marketed to address habituation, including closed-loop stimulation, multiple waveform libraries, and cascading or alternating micropulse paradigms. While these approaches are grounded in hypotheses about why habituation occurs, they remain largely heuristic, operating at the level of local spinal interactions rather than engaging the computational principles by which the brain interprets sensory input.

A potential mathematical bridge between habituation, the computational principles of the brain, and the delivery of neuromodulation therapy is variational free energy. The free energy principle, described by Friston, holds that any self-organizing system in equilibrium with its environment must minimize free energy (4). Here, free energy is an information-theoretic quantity, distinct from (but conceptually related to) the thermodynamic Gibbs free energy, which generally dictates the direction of spontaneous chemical reaction flow. At its core, free energy sets an upper bound on the “surprise” an organism experiences from sensory inputs, with surprise defined as the negative log probability of a sensory event. Intuitively, rare events are more surprising, since probabilities near zero yield large negative logarithms.

**Figure 1:**
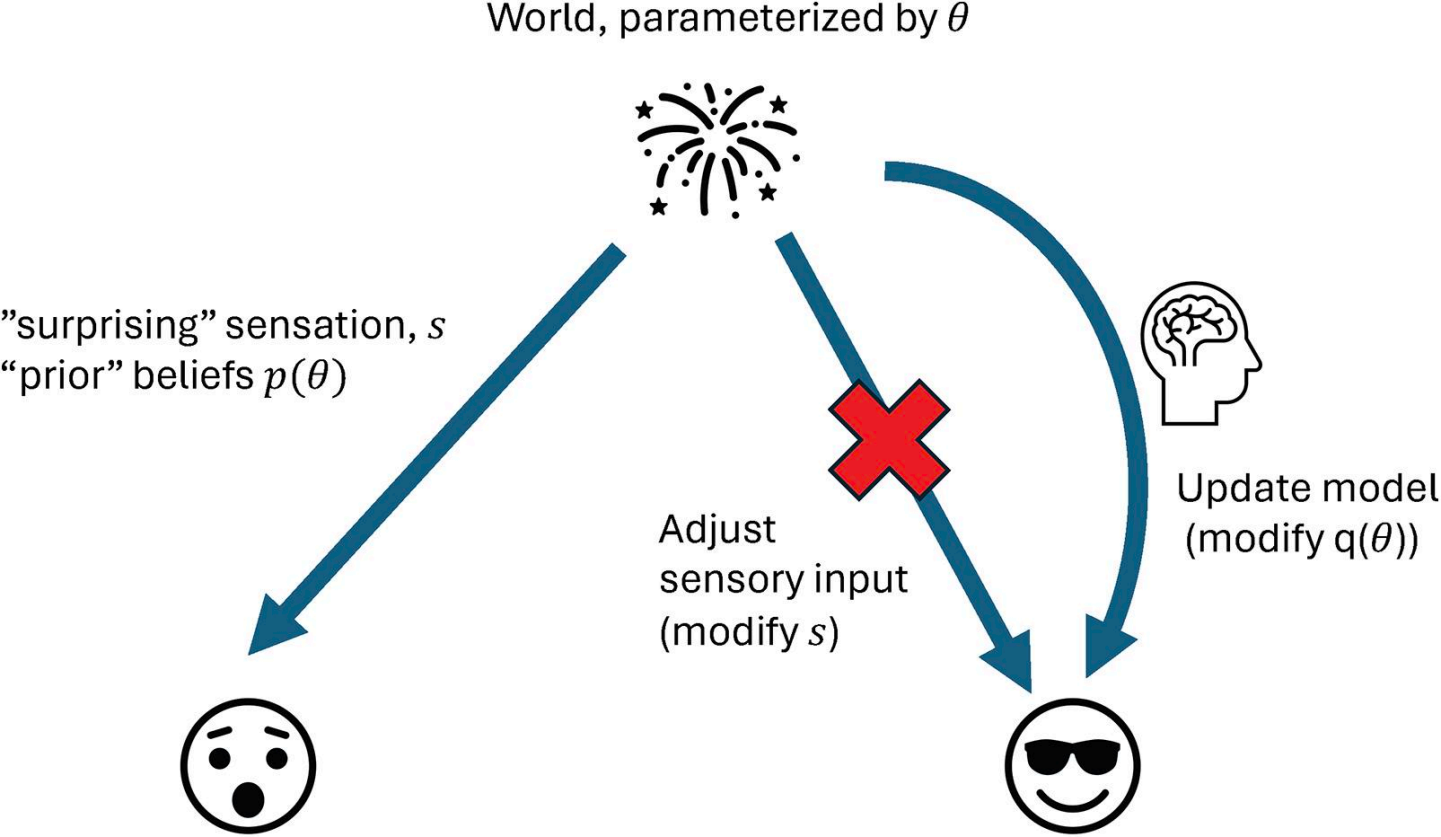
The brain as inference machine. Schematic of the brain and spinal cord as inference machines. Hidden world states (*θ*) generate sensory inputs (s). The brain may minimize free energy by either updating internal beliefs [recognition density q(*θ*)] to reduce prediction error (model update), or by altering sensory input to match expectations (active inference). This dual strategy illustrates perception vs. action as routes to restoring consistency between world and model.

The brain has two primary methods available to minimize free energy—it can update its model of the world so that previously rare events are incorporated in its model or it can seek sensory inputs to validate its internal model. For example, I may be surprised by explosions living in the suburbs but if one lives in a war zone, they may be less surprised by explosions, since their internal world model includes missiles. This is an example of model update. Contrarily, if noisy fireworks keep me awake at night on 4th of July, rather than updating my brain's model to always expect fireworks, it may be easier just to put in earplugs, thereby restoring sensory input that is consistent with my internal model (Figure 1).

Chronic pain itself can be understood as a high–free-energy attractor: once pain persists, the internal model assigns high prior probability to threat or damage, and ambiguous sensations are interpreted through that lens. SCS then adds an additional stream of sensory input. If that input is exogenous and stereotyped—consistent day after day, and only loosely coupled to the hidden state the brain is trying to infer—it can be explained away as predictable “device noise.” In VFE terms, the system can minimize free energy by reducing the precision assigned to stimulation-related sensory input rather than updating the pain model. That is a computational description of habituation. The rest of this paper builds an intuitive VFE toolbox and then applies it specifically to habituation and testable predictions relevant to neuromodulation.

Within this mathematical framework, the brain and spinal cord can be seen as inference machines that continually sample sensory data and minimize prediction error between incoming inputs and internal models. According to variational free energy, this process involves a tradeoff between accuracy (reducing prediction error) and complexity (the cost of updating the model). If the accumulated prediction error is sufficient to justify the cost, the system may transition between models—for example, from an “I’m in pain” worldview to an “I’m not in pain” worldview. If not, it may conserve the prior model and reduce prediction error by discounting or ignoring incoming data—for example, treating spinal cord stimulation as irrelevant noise.

Crucially, the statistical “distance” between models is not symmetric, and this asymmetry—formalized by the Kullback–Leibler (KL) divergence—means the cost of moving from one model to another depends on the direction of the update. In intuitive terms, driving from point A to B is not the same as driving from B to A. Applied to pain, this implies that it may be easier for the nervous system to fall back into a pain state than to climb out of one, even if prediction errors are equally reduced in both cases.

This review does not claim to fully mechanistically explain neuromodulation through free energy, but rather to offer an alternative computational lens on why therapies sometimes succeed and sometimes fail. Long-term failures, or reversion to pain states, will be framed within the free energy principle, with simple examples used to illustrate the mathematics. Some of the source materials for VFE are mathematically dense, which has likely limited generalizability for the clinical audience. While some math is unavoidable, the goal is to provide intuitive explanations for clinicians, with appendices and references available for readers seeking greater technical detail.

### Theoretical foundations

Let's describe sensory states (s)—what the patient feels—as being generated by hidden states of the world (*θ*, “theta”) that are not directly observed. For example, the sensation (s) “my foot is cold” could be explained by a cold floor, or by neuropathic dysesthesia after spine surgery; the brain is trying to infer which hidden cause (*θ*) is true. In general, the brain infers *θ* from s by combining prior beliefs with new data.

**Figure 2:**
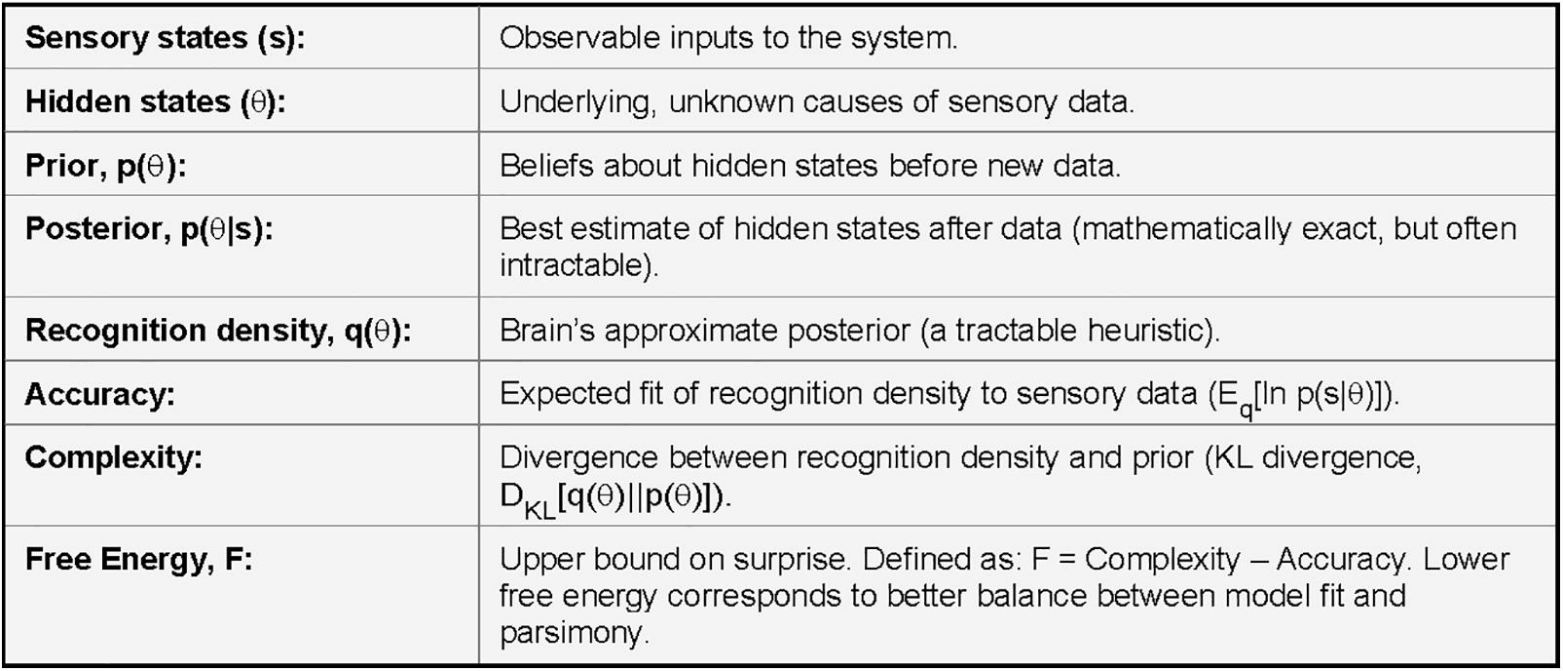
Box 1 — key definitions in variational free energy. Core terms used in the free energy framework.

To do this, we need to define some terms (listed in Figure 2). Note: terms are defined here as probability densities:

Recognition density, q(θ): the brain’s probabilistic *approximation* of what caused a sensation.

Posterior density, p(θs):the mathematically exact probability of hidden states given sensory input. This is the “gold standard,” but intractable for the brain to calculate directly.

Prior density, p(θ): beliefs about the world before seeing current data.

The distinction between these quantities can feel subtle. The recognition density is the brain's approximation to the posterior, because calculating the posterior directly with Bayes' theorem requires:

$$p(\theta s)=\frac{\;p(s\theta )\;p(\theta )}{\;p(s)}$$

where the computationally difficult term is the marginal likelihood:

p(s)=∫p(sθ)p(θ)dθ

Bayesian inference is simply probability updated by prior knowledge: posterior ∝ likelihood×prior. In this notation, p(s|*θ*) is the likelihood (how compatible a sensation is with a given hidden cause), p(*θ*) is the prior belief about causes, and p(s) is the “evidence” or marginal likelihood—how well the model, averaged over all possible *θ*, explains what was felt. Evaluating this integral would amount to a high-dimensional update over billions of possible hidden causes which is biologically implausible. Instead, we assume q(θ) is computed heuristically in a hierarchical system, with top-down predictions and bottom-up prediction errors interacting to reduce free energy. Moreover, this description applies to a *single update in time*. In a dynamic system, the recognition density from one moment becomes the prior for the next, seeding expectations forward.

**Figure 3:**
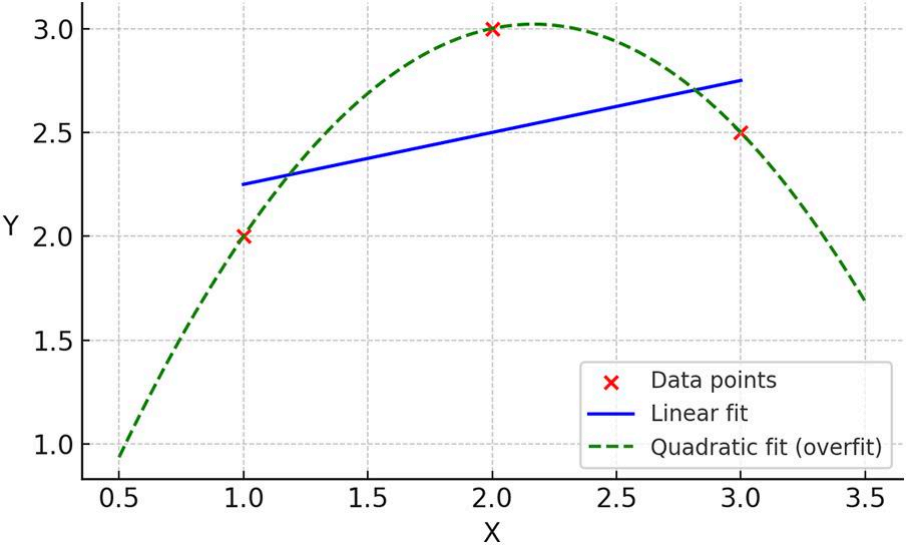
Overfitting analogy. Simple illustration of the tradeoff between accuracy and generalizability. A linear fit to three points leaves residual error but captures the underlying relationship, while a quadratic polynomial fits all points exactly yet risks poor generalization to new data. This analogy shows why free energy balances accuracy with complexity to avoid “overfitting” sensory experience.

A natural question is: why is free energy needed at all? That is, why can't the brain simply reduce prediction errors to zero? First, constant model updates would be metabolically costly. Secondly, this would lead to a well-known problem in machine learning called overfitting, where with enough parameters, we can fit any set of data exactly, but this fit may not be generalizable to future data. Imagine three points to which we are trying to fit a straight line. The straight line may fit them adequately, but a higher order equation, like a parabola, can fit them exactly. If the true phenomenon is linear, however, the parabolic model will extrapolate wildly and fail to generalize (Figure 3). The brain faces the same challenge — it must trade off accuracy against complexity to remain stable, efficient, and predictive.

With those foundations, in mind, let's look at the actual formulation of variational free energy:

“Surprise” can be defined as −log p(s): sensations that are improbable under the current model are more surprising. Variational free energy provides a computable quantity that upper-bounds this surprise:

$$F=\int q(\theta )\;\left(\frac{q(\theta )}{\;p(\theta s)}\right)d\theta -\ln p(s)$$

or

$$F=-\ln p(s)+\;D_{\mathrm{KL}}(q(\theta )\;||\;p(\theta s))$$

Because the KL divergence is always ≥ 0, free energy is always at least as large as surprise. Minimizing F therefore implicitly minimizes surprise by pushing q(*θ*) toward the true posterior *p*(*θ*|s), without requiring the brain to compute p(s) exactly.

In this formulation, Variational free energy, F, can be defined as the statistical difference between (Kullback-Leibler or KL divergence) of the brains internal model of the world (recognition density) and the “real” model of the world given our sensory input (posterior density or conditional density) plus the average surprise or entropy of the sensory input.

This can be rewritten as the trade-off between complexity and accuracy (for readers interested in the mathematical transformation, please see the excellent review by Friston (5).

$$F=D_{\mathrm{KL}}(q(\theta )\;||\;p(\theta ))-E_{q}[\ln p(s\theta )]$$

We will spend some time on this formulation as it has implications for the discussion on habituation. Here the KL divergence is the distance between the recognition density and prior density, which we will call complexity. We can look at it as the distance between two statistical distributions or the information lost be representing one model with the other. So in the setting of belief update, we can imagine this as the distance our internal model needs to travel from our prior beliefs to change our worldview. (In the prior formulation, the distance was between the recognition density and posterior density).

The $E_{q}[\ln p(s\theta )]\;$ term is the expected value over our recognition density (or weighted average) of the posterior density. Simply put this is the probability of receiving a sensory input given a certain parameter in the world, summed over our internal probabilities that those parameters exist. This term we will denote accuracy, which we can develop some intuition for as follows.

Illustrative Examples - Application to Habituation in Spinal Cord Stimulation:

Habituation as a VFE problem: In SCS, the key question is not only whether stimulation reduces pain intensity acutely, but whether the stimulation signal remains informative about the hidden state the brain is trying to infer. Open-loop stimulation can become highly predictable; once predicted, it generates little prediction error and can be treated as background. In VFE terms, the system reduces the precision assigned to the stimulation-related sensory stream and reverts to relying on priors and other sensory channels—often the entrenched pain model. Programming strategies used for habituation (cycling, alternating waveforms, closed-loop feedback) can be reframed as attempts to keep stimulation informative (i.e., to keep producing meaningful prediction error) rather than letting it become ignorable noise.

Imagine we live in a very simple world with two hidden parameters, $\theta_{1}$ and $\theta_{2}$, that generate sensory data *s*. Suppose the recognition density, q(θ), assigns probabilities $q(\theta_{1})=0.2$ and $q(\theta_{2})=0.8$. The posterior density, or the mathematical best guess about reality, p(s|θ), however, assigns likelihoods $p(s|\theta_{1})=0.7$ and $p(s|\theta_{2})=0.3$. The accuracy term will be:

$$E_{q}[\ln p(s|\theta )]=0.2\ln(0.7)+0.8\ln(0.3)\approx -1.03$$

Now suppose our recognition density is closer to the mathematically optimal distribution, with $q(\theta_{1})=0.6$ and $q(\theta_{2})=0.4$. In that case, the accuracy becomes:

$$E_{q}[\ln p(s|\theta )]=0.6\ln(0.7)+0.4\ln(0.3)\approx -0.695$$

which is a less negative, or more accurate, value. Given we subtract accuracy, the more accurate we are, the lower the free energy.

Let's look at a somewhat more relevant clinical example that may give us the “so what” of this mathematical exercise. Imagine a patient undergoing spinal cord stimulation for chronic pain. Prior to stimulation, they have a set of sensations, $s_{\mathrm{Pre}\text{-}\mathrm{Stim}},$ and a particular worldview, $p(\theta_{\mathrm{Pain}}),$ which represents a “prior” belief that the world is a painful place or that their body, which is part of the “world,” creates pain. After stimulation, their sensorium will change, and hopefully reduce the painful sensations, $s_{\mathrm{Post}\text{-}\mathrm{Stim}}$ Now we can formalize mathematically the competing processes which could lead to an update in a patient's mind that they no longer “recognize” the world as a painful place, $q(\theta_{\mathrm{NoPain}})$. This gives us two equations defining the free energy — $F_{\mathrm{Pain}}$, the free energy of remaining in a “pain” worldview, and $F_{\mathrm{NoPain}},$ an updated free energy from moving into a non-painful worldview.

$$F_{\mathrm{Pain}}=D_{\mathrm{KL}}(q(\theta_{\mathrm{Pain}})\;||\;p(\theta_{\mathrm{Pain}}))-\ln p(s_{\mathrm{Post}\text{-}\mathrm{Stim}}\theta_{\mathrm{Pain}}{)}_{q(\theta_{\mathrm{Pain}})}$$

and

$$F_{\mathrm{NoPain}}=D_{\mathrm{KL}}(q(\theta_{\mathrm{NoPain}})\;||\;p(\theta_{\mathrm{Pain}}))-\ln p(s_{\mathrm{Post}\text{-}\mathrm{Stim}}\theta_{\mathrm{NoPain}}{)}_{q(\theta_{\mathrm{NoPain}})}$$

Given that the brain works to reduce free energy, it will move into the not pain state only if the free energy is less. So if we subtract equation 1 from equation 2, and simplify $D_{\mathrm{KL}}(q(\theta_{\mathrm{Pain}})\;|\;p(\theta_{\mathrm{Pain}}))\;=\;0$ (distance between the pain belief set and itself is zero), we get:

$$\begin{array}{ccc} F_{\mathrm{NoPain}}-F_{\mathrm{Pain}}\; & =\; & D_{\mathrm{KL}}(q(\theta_{\mathrm{NoPain}})\;||\;p(\theta_{\mathrm{Pain}}))\;\\ \; & \; & -(\ln p{\left(s_{\mathrm{Post}-\mathrm{Stim}}\theta_{\mathrm{NoPain}}\right)}_{q(\theta_{\mathrm{NoPain}})}\;\\ \; & \; & -\ln p{\left(s_{\mathrm{Post}-\mathrm{Stim}}\theta_{\mathrm{Pain}}\right)}_{q(\theta_{\mathrm{Pain}})})\; \end{array}$$

**Figure 4:**
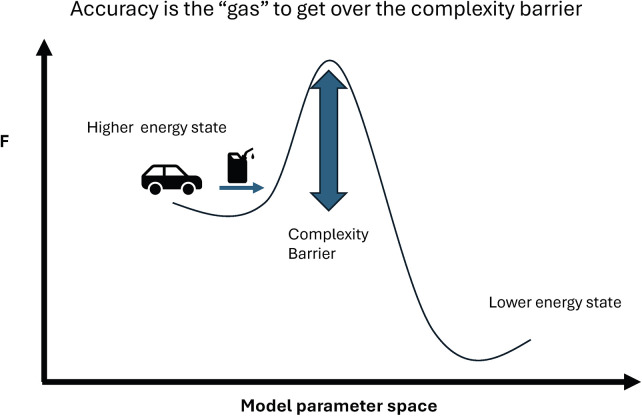
Complexity barrier. Free energy landscape metaphor. The “pain model” lies in a higher energy valley, while the “no-pain model” lies in a lower energy valley across a complexity barrier. Accuracy provides the “gas” to cross the hill, but insufficient prediction error relief may strand the system in the pain model. This illustrates how free energy constrains transitions between worldviews.

From this, we see that the difference in free energy is the distance between pain and non-pain beliefs minus the difference between accuracy of representing sensory input with a “non-painful” vs. “painful” worldview. Simply put, a patient will flip to a not-painful worldview only if the improvement in accuracy is enough to justify the distance between world representations (Figure 4).

This result has some valuable clinical implications. First, it may help explain why some patients appear “entrenched”—with disability, mood, sleep disruption, opioid dependence, and social withdrawal coupled to pain—and therefore show a smaller or less durable response to stimulation (6–10). In this case, the statistical “distance” to a non-painful world may be too far (normal qol and activity, social interactions, elimination of opioid use) even if spinal cord stimulation improves pain and is more accurately represented by a non-painful worldview. This also may explain why VAS scores do not always represent the overall satisfaction of patients with spinal cord stimulation therapy.

### Practical measurement note (VAS+QOL)

Clinically, we never observe the hidden state directly—we observe outcomes. A useful way to think about this is as an “outcome vector” rather than a single score: pain intensity (VAS), function (ODI or SF-36), quality of life (EQ-5D), mood (PHQ-9), sleep, and medication use. In VFE terms, therapy “works” when the brain's model better predicts this overall outcome profile over time—not when a single number moves. This is one reason VAS alone can be misleading: patients may report meaningful improvements in function and quality of life despite incomplete pain relief, and those gains may be more relevant to whether the system has truly transitioned into a stable low-pain state. This framing also sets up a mechanistic way to think about habituation: if stimulation changes only one component of the outcome profile, the broader model may not shift—or may drift back—once the stimulation signal becomes predictable.

As a corollary, it may also explain why there is more reversion to a non-responder state and “habituation” to therapy when multiple domains are affected which arises out of the asymmetric nature of the KL-divergence. That is, the distance out of a pain state may be further than the distance of falling back into pain. A concrete example will help with the mathematic abstraction. Imagine a four-parameter world of “medication use,” “functional status,” “psychological state,” and “physical pain.” The weight we give to each of the parameters will represent how much that domain explains our sensory state of the world. In our “painful” world probability density prior to spinal cord stimulation, lets imagine a uniform distribution where each of these we score as a 0.25, so our probabilities sum to 1. Meaning, medications, functional status, psychological state, and physical pain all contribute equally to the world view of being in pain. Now, let's say with spinal cord stimulation, the physical pain improves more significantly than the other domains, so it is more consistent with, and therefore, has an oversized weight on our new non-painful view of the world. Let's now give this a weighting of 0.4 and the rest of the domains a weighting of 0.2. The KL divergence, or distance from the “pain” worldview to “not pain” worldview, here will be calculated by:

$$D_{\mathrm{KL}}(q_{\mathrm{NoPain}}(\theta )||\;p_{\mathrm{Pain}}(\theta ))=\underset{\theta }{\sum }q_{\mathrm{NoPain}}(\theta )\ln\left(\frac{q_{\mathrm{NoPain}}(\theta )}{\;p_{\mathrm{Pain}}(\theta )}\right)$$

$$=0.2\ln\left(\frac{0.2}{0.25}\right)+0.2\ln\left(\frac{0.2}{0.25}\right)+0.2\ln\left(\frac{0.2}{0.25}\right)+0.4\ln\left(\frac{0.4}{0.25}\right)\approx 0.0541$$

Conversely, the reverse distance, from “not pain” back to “pain” worldview will be:

$$\begin{array}{rl} 0.25\ln\left(\frac{0.25}{0.2}\right) & +0.25\ln\left(\frac{0.25}{0.2}\right)+0.25\ln\left(\frac{0.25}{0.2}\right)\\ & \;+0.25\ln\left(\frac{0.25}{0.4}\right)\\ & \;\approx 0.0499 \end{array}$$

**Figure 5:**
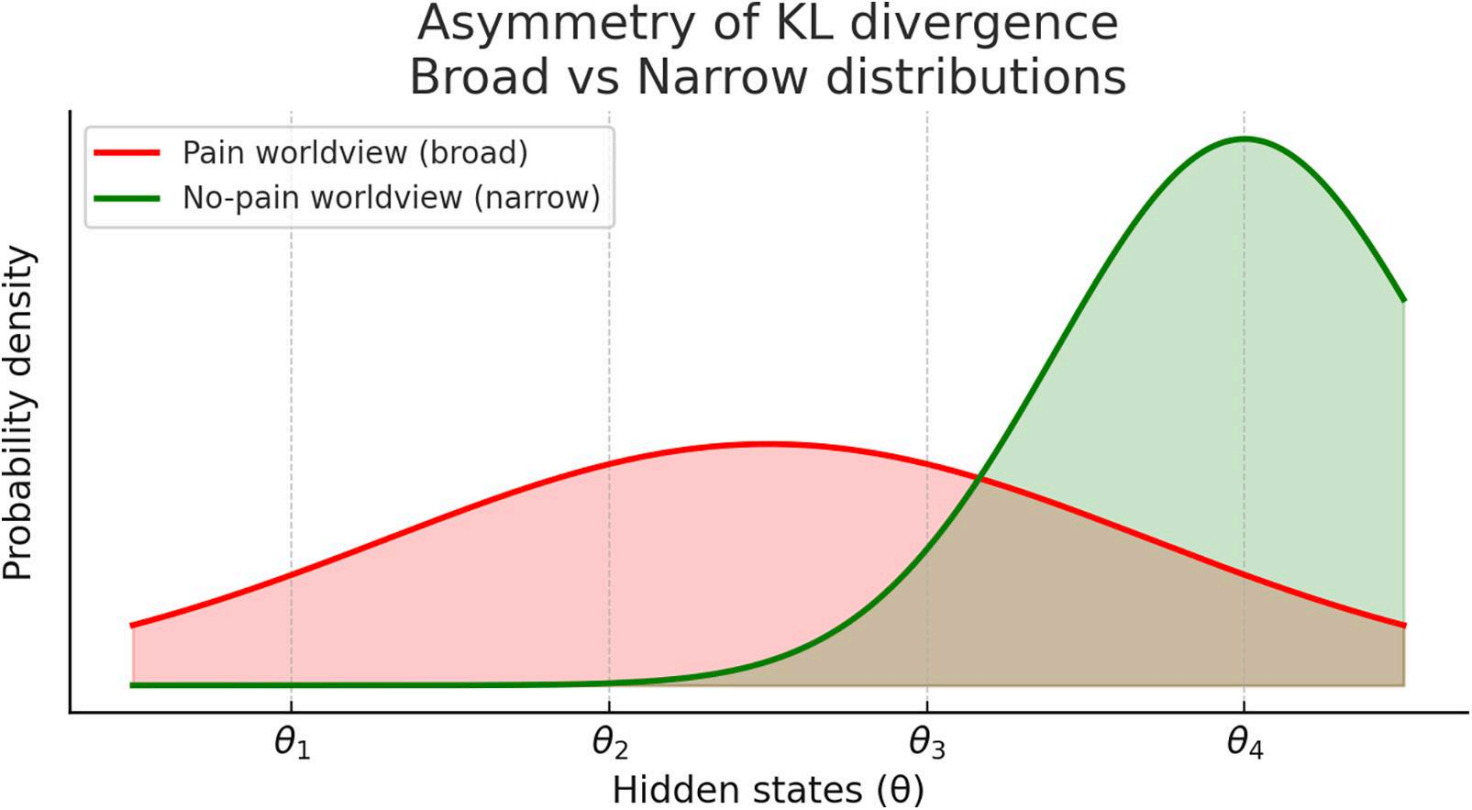
Asymmetry of Kullback–Leibler divergence. Stylized representation of probability densities as Gaussian curves. The “pain” model is broad across parameters, while the “no-pain” model is narrowly peaked at parameter 4 (e.g., physical pain improvement). KL divergence is asymmetric. Mathematically, it is more “costly” to represent a broad, high-variance distribution (the entrenched pain state) with a narrow, high-precision one (the relief state) because the narrow model fails to account for the “tails” of the broad model, resulting in significant information loss. This formalizes why it is computationally “easier” to revert to a broad pain state than to sustain a transition into a narrow relief state.

Meaning that the distance back into pain is shorter than the distance out of it, or it is sometimes easier to “fall off the wagon.” Figure 5 provides a stylized view of this asymmetry using Gaussian curves. The “pain” model is broad, representing a high-entropy state across multiple domains, while the “no pain” model is narrowly peaked, representing high-precision relief. Mathematically, it is more difficult to 'squeeze' a broad distribution into a narrow one, as much of the information in the “tails” is lost; this corresponds to the higher cost of achieving stable relief $D_{KL}\;\approx \;0.0541$. Conversely, it is easier for a narrow distribution to expand into a broader one, which represents the shorter statistical distance when reverting to a pain state $D_{KL}\;\approx \;0.0499$. This explains why the nervous system may “fall back” into pain more readily than it climbs out of it.

## Discussion

While this last example is somewhat contrived, it demonstrates that when too much of a therapy's value is concentrated in a single parameter (physical pain score improvement, for instance), it may become more fragile. This may explain why spinal cord stimulation may be more effective when used in conjunction with multimodality treatment including physical therapy, and why psychological factors are correlated with improvement after SCS (6–11). Similarly, it may explain increased efficacy of therapeutic waveforms that improve psychological or functional subdomains such as pain catastrophization or global impression of changes, or show changes on functional brain imaging (12–15).

This framework also offers a computational explanation for why certain interventions succeed by changing the *relevance* of pain rather than its intensity. For example, in cingulotomy, patients often report that they still “feel” the pain but are no longer “distressed” by it. In VFE terms, the intervention reduces the precision (or weight) assigned to the pain channel. Similarly, closed-loop SCS or primary cell batteries may succeed by reducing the patient's active engagement with the device, allowing the system to down-weight the “pain” parameter in the overall world model, thereby reversing the KL-divergence relationship and making the pain state harder to re-enter.

There may be more phenomena and explanations that can be elucidated and formalized by this framework, and my review is not meant to be exhaustive. Possibly there may be fertile ground in evaluating other implications of the free energy framework, such as how the brain and spinal cord may encode precision with relation to an SCS waveform. For instance, is the quest for more consistent stimulation more likely to lead to consistent therapeutic response as the brain sees it as a meaningful feature of the world, or is it more likely to be seen as background noise, especially if the pain signals remain salient. Alternatively, would alternating waveforms lead to less habituation, or would this simply relegate the SCS signal to the category of a non-useful source of sensory information. My own suspicion is that it depends on whether stimulation provides a meaningful therapeutic, multidomain response, or whether it is just extra noise.

Finally, there may be risks of conflating the free energy mathematical framework with mechanistic action of spinal cord stimulation. For instance, I do not fundamentally believe that manipulating the information-theoretic quantities of the SCS signal itself is likely to make for a more effective spinal cord stimulator, and I am not advocating for the creation of “higher entropy” SCS waveforms. With that in mind, future areas of research may evaluate changes in ECAPs, beta-band LFPs, ERNAs, or other responsive signals to determine if those signal characteristics changing over time may inform us of effective model update to a “therapy-enhanced” worldview or may be early signals of reversion or habituation.

With that in mind, the variational free energy framework may give several empirically testable hypotheses for neuromodulation. If habituation represents a drift toward less adaptive inference, measurable correlates may include reduced variability, complexity, or dynamic range in neural and behavioral signals over time. Conversely, stimulation paradigms that maintain benefit may preserve rich, metastable dynamics that support continuous model updating. These ideas could be examined through longitudinal analyses of electrophysiologic or behavioral metrics—such as variability in evoked compound action potentials (ECAPs), changes in oscillatory diversity on EEG, or broader functional measures of adaptability. While these markers do not measure variational free energy directly, they could serve as observable proxies of the brain's capacity for flexible inference and model updating. Future studies could test whether sustained therapeutic benefit corresponds to maintaining this adaptive regime rather than converging toward overly stable or predictable neural states.

In summary, variational free energy is an information theoretic quantity which provides a mathematical framework for how the brain represents the world. The emergent properties of this framework may inform clinical dilemmas in neuromodulation.

## Data Availability

The original contributions presented in the study are included in the article/Supplementary Material, further inquiries can be directed to the corresponding author.
